# Insights into Cancer Patients’ Experiences and Needs in the Northeast Region of India: A Qualitative Study

**DOI:** 10.3390/healthcare13212748

**Published:** 2025-10-30

**Authors:** Redolen Rose Dhar, Reshmi Bhageerathy, Ramesh Holla, Anisha Mawlong

**Affiliations:** 1 Department of Health Information Management, Manipal College of Health Professions (MCHP), Manipal Academy of Higher Education, Manipal 576104, India; redolen.dhar1@learner.manipal.edu; 2 Department of Community Medicine, Kasturba Medical College Mangalore, Manipal Academy of Higher Education, Manipal 575001, India; ramesh.holla@manipal.edu; 3 Department of Radiation Oncology, Civil Hospital, Shillong 79300, India; anishamawlong@gmail.com

**Keywords:** cancer, cost communication, financial burden, out-of-pocket expenditure, tribal population, universal health coverage

## Abstract

**Background/Objectives**: Cancer remains a major public health concern in India, with the Northeast Region (NER) reporting the country’s highest incidence rates. In Meghalaya, a predominantly tribal state, cultural beliefs, financial hardship, and limited healthcare access significantly affect cancer diagnosis and treatment outcomes. This study explores the experiences and needs of cancer patients in Meghalaya, India, to inform culturally sensitive, patient-centred, and financially inclusive approaches to cancer care among tribal populations. **Methods**: A qualitative study was conducted among 19 participants (12 patients and 7 caregivers; in cases where patients were unable to communicate effectively due to physical weakness or treatment-related complications, their primary caregivers, those directly linked to the specific patients, were interviewed instead) receiving treatment at Civil Hospital, Shillong, between August and November 2023. In-depth interviews were conducted in Khasi, translated into English, and analysed thematically following COREQ guidelines. **Results**: Ten key themes emerged. Patients often attributed early symptoms to supernatural causes and sought traditional healers, delaying diagnosis. Many experienced fragmented care pathways, misinformation, and fear of treatment side effects. The financial burden was severe, with high out-of-pocket costs for travel, diagnostics, and medicines, despite partial relief through the Meghalaya Health Insurance Scheme. Communication about costs between patients and providers was limited, leaving families unprepared for the expenses. Emotional distress, loss of livelihood, and dependence on family support were common, while faith and spirituality served as major coping mechanisms. **Conclusions**: Cancer care in Meghalaya is shaped by intertwined cultural, economic, and systemic barriers. Strengthening culturally tailored health education, decentralised diagnostic services, structured financial counselling, and cost transparency can improve care delivery. Future research should adopt multi-centre, longitudinal approaches to guide equitable, patient-centred cancer policies in tribal and rural settings.

## 1. Introduction

Cancer remains a major global health challenge, accounting for nearly one in six deaths worldwide [1]. In India, the burden of cancer is steadily increasing, with marked regional disparities in incidence and outcomes [2]. According to the Indian Council of Medical Research (ICMR), NER consistently reports the highest incidence of cancer in the country [3]. The NER comprising Arunachal Pradesh, Assam, Manipur, Meghalaya, Mizoram, Nagaland, Sikkim, and Tripura has a predominantly tribal population, with tribal communities forming the majority in Mizoram, Nagaland, Meghalaya, and Arunachal Pradesh [4]. These tribal communities are characterised by distinct socio-cultural traditions, spiritual worldviews, and indigenous interpretations of illness that influence their health-seeking behaviour [5,6]. In Meghalaya and Assam, cancer symptoms are often perceived as the result of poisoning (*bih*) or supernatural afflictions (*skai*), leading individuals to seek care from traditional healers locally known as *dawaiduna* or *ojha* before consulting biomedical providers [5,7,8]. Such culturally embedded beliefs contribute to delays in diagnosis and treatment, leading to late-stage presentations and poorer outcomes.

Beyond these cultural and behavioural influences, the region’s cancer burden is evident in epidemiological data. Population-based cancer registries report that the NER carries a disproportionate share of the national burden. Aizawl district in Mizoram records the highest age-adjusted incidence rate (AAR) among males (269.4 per 100,000), while Papum Pare in Arunachal Pradesh reports the highest AAR among females (219.8 per 100,000). In contrast, AARs in metropolitan registries such as Delhi (147.0 per 100,000 in males) and Bengaluru (146.8 per 100,000 in females) are considerably lower [8]. These figures highlight the combined effects of lifestyle, environmental, and health system factors contributing to the region’s elevated incidence. Systemic and economic constraints further exacerbate the challenges of cancer care in the NER. Healthcare infrastructure remains underdeveloped, particularly in rural and tribal areas, where over 80% of the population resides [3]. Many districts lack cancer treatment centres, diagnostic facilities, and trained oncology personnel, compelling patients to travel long distances, often outside the region, to access services [9]. This travel imposes logistical difficulties and substantial emotional and financial strain, with limited insurance coverage and high out-of-pocket costs forcing families to borrow or sell assets to finance treatment [6,9,10].

Amid these systemic and financial barriers, upper digestive tract cancers, including oesophageal, stomach, and hypopharyngeal cancers, are especially prevalent in the region [11]. These patterns are associated with lifestyle practices such as the consumption of tobacco, alcohol, betel nut, smoked meat, and spicy foods, all of which are established carcinogenic risk factors [3,12]. Despite this high risk, awareness and screening uptake remain low, hindered by poor health literacy, cultural stigma, and inadequate outreach [13,14,15].

Although national programmes such as the National Programme for Prevention and Control of Cancer, Diabetes, Cardiovascular Diseases, and Stroke (NPCDCS) have strengthened service delivery, tribal and rural populations in the NER continue to experience inequitable access to care. Geographic isolation, cultural marginalisation, and sustained underinvestment in health infrastructure perpetuate disparities in diagnosis, treatment, and survival [16,17,18]. Moreover, existing health communication strategies often overlook indigenous worldviews, thereby limiting their effectiveness and community engagement [19]. Given these multifaceted challenges, there is an urgent need to understand how socio-cultural, financial, and health system factors intersect to influence the lived experiences of cancer patients in the NER. This study explores the experiences and needs of cancer patients in Meghalaya, India, to inform culturally sensitive, patient-centred, and financially inclusive approaches to cancer care among tribal populations.

## 2. Materials and Methods

### 2.1. Study Setting

This qualitative study was conducted among individuals diagnosed with cancer who were receiving treatment at Civil Hospital, Shillong, the primary state-run public referral hospital for cancer care in Meghalaya. As the main referral centre, Civil Hospital Shillong serves patients from various districts across the state, encompassing both urban and rural populations, and reflects a diverse range of tribal and socio-economic backgrounds. The study was conducted between August 2023 and November 2023, following ethical clearance from the Kasturba Hospital Institutional Ethics Committee, Karnataka, and the Pasteur Institute Ethics Committee, Shillong, Meghalaya. Informed consent was obtained from all participants after they were provided with detailed information regarding the study objectives, procedures, and their rights, as outlined in the participant information sheet.

### 2.2. Study Design and Data Collection

A qualitative design employing purposive sampling was used to explore the experiences and needs of individuals affected by cancer and their caregivers. The participants included patients diagnosed with Head and Neck (HN), Respiratory (RP), or Gastrointestinal (GI) cancers, ranging from Stage I to Stage IV, who had initiated or were undergoing treatment at Civil Hospital, Shillong. These specific cancer types were selected because they represent the most common tobacco-related cancers in Meghalaya and together account for the top five cancer sites in the state, as reported by the ICMR [2]. Including these cancers ensured that the study captured the lived experiences of those most affected by the predominant regional cancer burden. In cases where patients were unable to communicate effectively due to physical weakness or treatment-related complications, their primary caregivers, those directly linked to the specific patients, were interviewed instead. No independent or unrelated caregivers were included. The study adhered to the Consolidated Criteria for Reporting Qualitative Research (COREQ) guidelines [20] to ensure rigour and transparency throughout the research process.

Prior permissions were obtained from the Directorate of Health Services and the Medical Superintendent of Civil Hospital, Shillong, as required by the respective ethics committees. Access to hospital records was granted to identify potential participants. With the assistance of hospital records staff, contact details were retrieved, and participants were approached during their follow-up visits to the hospital’s outpatient department (OPD). After a detailed explanation of the study and written informed consent, the primary researcher (the first author), trained in qualitative methods, conducted interviews in a designated waiting area within the hospital to minimise disruption of treatment schedules. With a professional background in nursing and public health, the primary researcher had an informed understanding of patient care processes and the functioning of health systems, which facilitated effective engagement and communication during interviews. Awareness of potential preconceptions regarding healthcare access and patient vulnerability was maintained throughout the study. To enhance reflexivity and minimise bias, several strategies were implemented, including pilot testing of the interview guide, maintaining a reflexive journal to record observations and assumptions, and conducting regular debriefing sessions with the research team to ensure analytical rigour and neutrality.

A total of 21 individuals were approached, of whom 19 agreed to participate (12 patients and 7 caregivers). Two participants who initially consented withdrew due to urgent travel. Each participant was interviewed once, and no repeat interviews were conducted. In-depth interviews were conducted using a semi-structured interview schedule, which was validated by experts in oncology, public health, health information, and social work. The panel assessed the content validity, clarity, and relevance of the guide; feedback was incorporated to refine and align it with the study objectives. Interviews were conducted in Khasi, the local language, and later translated into English. Each interview lasted approximately 30 to 40 min. Thematic saturation was considered achieved when no new codes or concepts emerged in two consecutive interviews (following the 17th interview); two additional interviews were conducted thereafter to confirm the saturation.

### 2.3. Analysis

Data were analysed using a thematic content analysis approach [21], which is widely used for identifying and interpreting patterns in qualitative data. All audio recordings were first transcribed verbatim in Khasi and then translated into English for analysis. To ensure translation accuracy and cultural fidelity, two bilingual Khasi–English experts independently reviewed the translated transcripts. Discrepancies were discussed collaboratively until consensus was reached. A subset of transcripts was back-translated into Khasi to verify semantic and contextual equivalence, and Khasi language experts were consulted to preserve the meaning of culturally specific expressions and idioms. An inductive coding approach was adopted [22], allowing codes and themes to emerge organically from the data. To establish intercoder reliability, 35% of the transcripts were independently double-coded by two researchers (RRD and BR). Coding discrepancies were identified through cross-comparison, discussed jointly, and resolved through consensus. In instances where disagreement persisted, a third senior researcher (RH) reviewed the excerpts to facilitate adjudication and ensure consistency. Intercoder reliability was calculated using the percentage agreement (PA), which yielded a score of 90%, indicating a high level of concordance and coding consistency across researchers. A codebook was developed and refined iteratively, and a framework matrix was applied using Microsoft Excel (version 16.6) to organise themes and subthemes systematically. MAXQDA 2022 software was used to efficiently manage and analyse the data.

Each quotation presented in the results section is accompanied by a unique identifier that includes the cancer type, age, sex, and participant role. For example, (HN, 34 y, Male, Patient) refers to a 34-year-old male patient with head and neck cancer, and (GI, 48 y, Female, Caregiver) denotes a female caregiver of a patient with gastrointestinal cancer. All caregivers interviewed were directly associated with the patients represented in this study; no independent caregiver–patient pairs were included. Analytical rigour was maintained through independent coding, intercoder verification, and audit trails documenting analytic decisions. The study adhered to the COREQ 32-item checklist to ensure methodological transparency, credibility, and rigour.

## 3. Results

**Figure 1:**
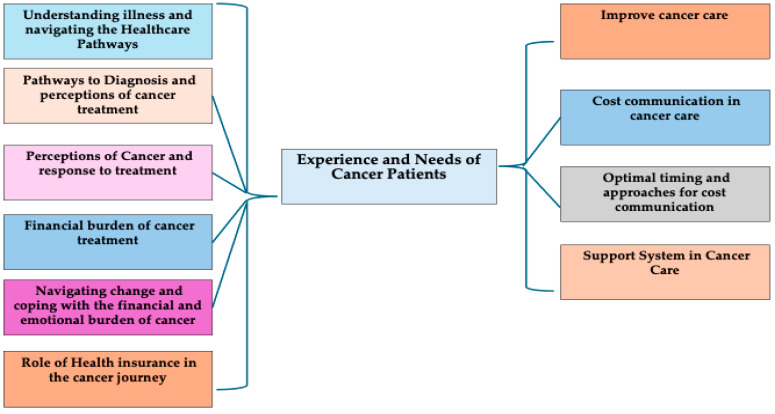
Experience and needs of cancer patients in Meghalaya.

Table 1 presents the demographic characteristics and distribution of cancer types among the study participants. The variables include age, gender, religion, marital status, literacy, occupation, and residence. The study included nearly equal participants from different age groups, with a majority of male patients who identified as Christian, reflecting the demographics of this Christian-majority state. Most participants (approximately 90%) had an education level up to secondary school and resided in rural areas. Occupations varied, with almost half engaged in daily wage work. More than half were diagnosed with gastrointestinal cancers, followed by head and neck cancers. The results are presented under ten themes that reflect the experiences and needs of cancer patients, as shown in Figure 1.

### 3.1. Experiences of Cancer Patients

#### 3.1.1. Understanding Illness and Navigating the Healthcare Pathway

The analysis of patient narratives reveals how cultural beliefs and traditional interpretations shape health-seeking behaviours. Many participants initially attributed symptoms to supernatural causes such as *“kymbat” (black magic)* and sought traditional healers before turning to biomedical care.

*“My illness began in 2016 when I could feel something inside my throat… I thought it was kymbat, so I went to a traditional healer.”* (GI, 53 y, male, patient)

The transition to formal care often occurred only after symptoms worsened, leading to delays and misdiagnoses. These experiences highlight gaps in early detection and community awareness among both patients and frontline healthcare providers.

*“At first, there was swelling on my neck; they told me it was a thyroid issue… Later, it was confirmed as cancer.”* (HN, 48 y, male, patient)

#### 3.1.2. Pathways to Diagnosis and Perceptions of Cancer Treatment

Many participants first relied on traditional medicine before seeking formal care, reflecting the coexistence of cultural and biomedical systems. As symptoms persisted, they navigated multiple healthcare facilities, often private hospitals for preliminary testing and government hospitals for treatment, highlighting logistical and financial challenges they faced.

*“I went to one private hospital… then another… before finally being referred to a government hospital where cancer was confirmed.”* (GI, 42 y, male, patient)

Reactions to diagnosis ranged from fear and sadness to reassurance through faith and trust in doctors. Participants expressed appreciation for empathetic communication and competence among healthcare providers but reported challenges related to incomplete information and medication shortages.

*“The doctor explained everything clearly and encouraged me to start treatment immediately. That gave me courage.”* (GI, 57 y, female, patient)

#### 3.1.3. Perceptions of Cancer and Responses to Treatment

Participants’ perceptions reflected a mixture of fear, hope, and misinformation. Traditional remedies (*dawai kymbat*) were widely used, often due to peer influence or fear of side effects. Some believed that biomedical treatment could worsen their condition.

*“People told me if I take treatment here, I will face side effects like hair fall… so I was hesitant.”* (RP, 62 y, male, patient)

Many considered cancer unaffordable and incurable until informed otherwise by doctors. Awareness of financial assistance and health schemes shifted perceptions positively, reinforcing the importance of patient education and effective communication between providers.

*“We always thought cancer treatment was too expensive, but later we learned that help was available.”* (HN, 50 y, male, caregiver)

#### 3.1.4. Financial Burden of Cancer Treatment

Cancer imposed substantial financial, emotional, and social strain on families. Direct and indirect costs, such as travel, accommodation, and food, were burdensome, especially for rural patients.

*“If we hire a car, it costs more than ₹4000, plus food and travel expenses are higher than the treatment.”* (RP, 53 y, male, patient)

The Meghalaya Health Insurance Scheme *(MHIS)*, a public health insurance scheme introduced by the government of Meghalaya, is locally referred to as the “*smart card*” and provides partial coverage for hospitalisation costs. However, patients reported that outpatient care, diagnostics, and medicines often required out-of-pocket payments.

*“Smart cards only help if you’re admitted. For weekly medicines, it costs around ₹8000.”* (GI, 45 y, male, patient)

These narratives reveal psychological distress due to financial uncertainty and reliance on borrowing or informal support.

*“We borrowed money from relatives every time we came here. None of the tests were available locally.”* (HN, 46 y, male, patient)

#### 3.1.5. Navigating Change and Coping with Financial and Emotional Burden

Participants described significant lifestyle disruptions, role changes, and emotional exhaustion following a diagnosis. Families often pooled resources, sacrificed savings, and relied on spiritual faith to cope with uncertainty.

*“My family borrowed money to help me… I can’t work right now; we live on loans.”* (RP, 42 y, female, patient)

*“We just have to keep faith in God and pray every day.”* (GI, 51 y, male, caregiver)

These accounts emphasise the interconnection between economic hardship, caregiving strain, and emotional well-being in the cancer experience.

#### 3.1.6. Role of Health Insurance in the Cancer Journey

Health insurance through the *MHIS* (“*smart card*”) reduced hospitalisation costs but did not cover outpatient services, leading to mixed perceptions of its usefulness.

*“Before the smart card, I felt it was expensive, but now it is affordable.”* (GI, 42 y, male, patient)

Some participants lacked awareness of how to utilise their insurance benefits, relying on family members to manage them, underscoring the need for health literacy and system navigation support.

*“I have the smart card, but my family handles everything. I don’t know how it works.”* (HN, 45 y, male, patient)

### 3.2. Needs of Cancer Patients

#### 3.2.1. Improving Cancer Care

Participants called for behavioural change, improved awareness, and systemic strengthening to address the growing cancer burden.

*“We need to understand lifestyle factors… behavioural change is needed among both the public and healthcare staff.”* (HN, 50 y, male, caregiver)

They also emphasised mental health support and flexible service schedules to improve patient comfort and continuity of care.

*“It’s too cold at 8:30 a.m. for chemotherapy and radiation therapy… timings should consider patient comfort.”* (HN, 50 y, male, caregiver)

#### 3.2.2. Cost Communication in Cancer Care

Patient narratives revealed that discussions about treatment costs were often limited or absent during consultations. Many participants reported not receiving clear information about expected expenses or available financial support. This lack of communication often left families unprepared for unforeseen costs.

*“They have never discussed what to expect about our expenses. So, we have to decide for ourselves.”* (HN, 39 y, male, caregiver)

*“The doctor just wrote the tests and told me these were the tests that needed to be done.”* (HN, 46 y, male, patient)

A few participants mentioned doctors who briefly discussed treatment costs, but such conversations were inconsistent and mostly occurred only after treatment began.

*“It would be helpful if the doctor informed patients about the cost beforehand.”* (GI, 53 y, male, patient)

Participants also described barriers, including a lack of time during consultations, insufficient guidance, and confusion about insurance coverage.

*“Before going to Guwahati (a state name), we asked if we could use the smart card, but nobody gave us proper information.”* (HN, 42 y, male, patient)

The findings suggest that many health professionals may not be familiar with detailed treatment costs, which makes it difficult for them to provide patients with adequate guidance. Designated financial counsellors or hospital cost experts could assist in this process. Moreover, communicating total treatment costs is challenging due to variations in cancer types, disease stages, and individual responses to therapy. These uncertainties often make it hard for patients to plan financially and may even lead some to discontinue or avoid treatment.

#### 3.2.3. Optimal Timing and Approaches for Cost Communication

Participants preferred discussing costs before starting treatment, enabling them to make informed decisions and ensure financial preparedness. They also valued continuous cost guidance throughout the treatment process.

*“It’s important to know before treatment how much we’ll need to arrange.”* (GI, 45 y, male, patient)

Cost communication should thus be an ongoing, collaborative process involving both providers and patients, rather than a one-time interaction.

#### 3.2.4. Support Systems in Cancer Care

Family and faith emerged as the most vital support systems. Families provided motivation, financial help, and emotional comfort, while spirituality offered hope and resilience.

*“My family encouraged me to go for treatment and told me to have faith in God.”* (GI, 55 y, male, patient)

*“We have to look upon Him and let Him guide us.”* (GI, 47 y, male, patient)

These reflections highlight the holistic nature of cancer care, where emotional, spiritual, and social supports are integral to recovery.

## 4. Discussion

This study provides rich, context-specific insights into the lived experiences and unmet needs of cancer patients in Meghalaya, a predominantly tribal state in Northeast India. The findings reveal how deeply embedded cultural beliefs, systemic healthcare gaps, and inadequate communication jointly shape the cancer care journey, echoing broader patterns observed across India and other countries. Similar to findings from the other part of India, where cancer is often perceived as a spiritual affliction or a consequence of karma [23], participants in our study attributed early symptoms to supernatural causes, such as *kymbat* (Black Magic), and sought traditional remedies before engaging with formal healthcare systems. These culturally rooted interpretations parallel evidence from sub-Saharan Africa, where cancer has been viewed as the result of curses or spiritual punishment, leading to delayed medical care [24]. Within the Khasi community, the blending of Christian faith and indigenous traditions created a complex belief system that simultaneously provided coping strength and contributed to delayed health-seeking. Similar syncretic patterns have been observed in different countries, where faith and biomedical care coexist within pluralistic healing systems [25,26,27]. Such cross-contextual findings underscore the importance of culturally sensitive health education and community engagement strategies that incorporate traditional healers and faith leaders into referral networks, aiming to promote early diagnosis and mitigate misconceptions [5,28].

The prolonged diagnostic journeys reported by patients in this study, characterised by multiple referrals, misinterpretations of symptoms, and delayed diagnoses, reflect broader systemic challenges in India and other resource-constrained settings. Similar patterns have been documented in Indian states such as Assam and Sikkim, where inadequate diagnostic infrastructure and limited specialist availability hinder timely care [6,29]. International evidence corroborates these findings: fragmented referral pathways and weak diagnostic systems have been linked to the advanced stage of presentation at diagnosis [30,31]. Establishing referral-integrated “hub-and-spoke” models that connect district hospitals with tertiary centres, as demonstrated successfully in other LMICs such as Brazil and South Africa, could streamline diagnostic processes and strengthen cancer care delivery in Meghalaya [32,33].

The study also underscores the multidimensional financial distress experienced by patients and their families. Despite support through the Megha Health Insurance Scheme (MHIS), many participants incurred high out-of-pocket expenses (OOPEs) for medicines, diagnostics, and transportation, a finding that mirrors those from other Indian states, where publicly financed insurance schemes inadequately cover outpatient and ancillary costs [34,35]. This aligns with global data showing that cancer patients in LMICs frequently face catastrophic healthcare spending, often exacerbated by travel and accommodation costs [36]. In geographically isolated regions, such as Meghalaya, the indirect costs of accessing care can exceed direct treatment costs, a trend also observed in other countries [37,38,39]. Innovative solutions, such as tele-oncology and mobile screening units, which have been successfully implemented in other countries, offer promising models for reducing these indirect expenses. This support enables them to travel without financial difficulty, encouraging them to persist with their treatment [40,41,42].

A novel contribution of this study lies in identifying cost communication as a critical yet neglected aspect of patient-centred oncology care. Participants expressed a strong desire to discuss treatment costs before starting therapy, allowing them to prepare financially and avoid unexpected burdens. However, such discussions were rare due to time constraints, overcrowded clinics, and patients’ reluctance to question medical authority. Similar gaps have been reported internationally, where fewer than one-third of oncologists routinely discuss costs despite strong patient preference for transparency [43,44]. These findings emphasise the importance of institutionalising structured financial counselling and incorporating cost communication into routine cancer consultations. Clear communication about costs has been shown to improve patient preparedness, foster trust, and enhance adherence to treatment [45,46]. Addressing cost transparency at the policy level through inclusion in national oncology guidelines and hospital protocols could significantly mitigate financial toxicity for cancer patients in India.

Beyond the financial and systemic barriers, the emotional landscape of cancer care in Meghalaya was profoundly shaped by family support, faith, and spiritual coping. Patients drew strength from their families and religious beliefs to navigate the psychological burden of the illness, a finding consistent with both Indian and international studies demonstrating that the buffering effect of spirituality and social support [47,48]. Nevertheless, reliance on spiritual coping should not substitute for formal psychosocial support, which remains underdeveloped in Indian cancer centres [49]. Integrating culturally appropriate psychosocial interventions, such as task-shared counselling and community-based support, can enhance the overall quality of life and emotional well-being of patients.

Collectively, these findings highlight the urgent need for multi-pronged interventions in Meghalaya’s cancer care ecosystem. Policies should promote culturally tailored health education, strengthen decentralised diagnostics through hub-and-spoke models, expand financial protection to cover indirect costs, and institutionalise cost communication as part of patient-centred oncology care. These recommendations align with the goals of the National Programme for Prevention and Control of Cancer, Diabetes, Cardiovascular Diseases and Stroke (NPCDCS), Ministry of Health and Family Welfare, 2021, and reflect international calls for equitable, patient-centred cancer systems in LMICs [50,51]. By situating the experiences of cancer patients in Meghalaya within both national and global contexts, this study contributes to the growing body of evidence emphasising the integration of cultural sensitivity, financial transparency, and psychosocial support into cancer care delivery in resource-limited and culturally diverse settings.

### Study Limitations

This study has several limitations that should be taken into account when interpreting the findings. The study employed a purposive sampling approach, which is suitable for exploratory qualitative research but limits the statistical generalisability of the findings to other populations. It was conducted in a single public tertiary hospital in Meghalaya, focusing on a specific group of cancer patients; therefore, the results may not fully represent the experiences of individuals seeking care in private or out-of-state cancer centres. The absence of long-term follow-up restricted the ability to capture how patients’ perceptions, coping mechanisms, and financial circumstances evolved over time. As with most qualitative research, there is a possibility of social desirability bias, where participants may have provided responses they believed were expected or socially acceptable, particularly regarding their trust in doctors and satisfaction with the care they received. Although interviews were conducted in Khasi and translated into English with cross-checking to ensure accuracy, some linguistic nuances and cultural meanings may have been lost in translation, which could potentially influence data interpretation.

Member checking was not conducted due to logistical constraints and the clinical condition of some participants during the data collection process. Nevertheless, credibility was strengthened through strategies such as intercoder reliability checks, reflexive journaling, and team-based consensus discussions. These methodological limitations may influence the transferability and external validity of the findings; however, the study provides contextually rich, in-depth insights into the lived experiences of cancer patients in a tribal, resource-limited setting. Future research incorporating multi-centre samples, longitudinal follow-up, and mixed-method designs would enhance the robustness and applicability of the evidence across diverse contexts.

## 5. Conclusions

This study underscores how cultural beliefs, financial hardship, and systemic healthcare barriers collectively shape the cancer care experiences of patients in Meghalaya, India. Deep-rooted indigenous worldviews and limited health literacy delay diagnosis, while high out-of-pocket costs and inadequate insurance coverage intensify financial distress. Strengthening cost communication within oncology care is vital to support informed and financially prepared decision-making. Culturally sensitive health education, community engagement with traditional and faith-based leaders, and decentralised diagnostic networks can enhance early detection and access to care. Policymakers should prioritise expanding financial protection to include indirect costs and integrating psychosocial and financial counselling into cancer services. Future research should adopt multi-centre and longitudinal designs to examine how cultural, economic, and systemic factors influence cancer care across diverse tribal and rural contexts in India.

## Figures and Tables

**Table 1:** Demographic characteristics of the participants.

Variables	Category	Counts	Percentage (%)
Age	≥50	10	52.6
	<50	9	47.4
Gender	Male	12	63.2
	Female	7	36.8
Religion	Christian	19	100
Marital Status	Married	17	89.5
	Unmarried	2	10.5
Literacy Status	No formal education	5	26.3
	Primary	7	36.9
	Secondary	3	15.9
	Highschool	2	10.5
	Graduate	2	10.5
Occupation	Business	5	26.2
	Farmer	4	21.1
	Daily wage labor	8	42.1
	Driver	1	5.3
	Engineer	1	5.3
Residence	Urban	3	15.8
	Rural	16	84.2
Cancer	Gastrointestinal Cancer	10	52.6
	Respiratory Cancer	3	15.8
	Head and Neck Cancer	6	31.6

*Note*: Percentages were calculated within each variable and standardised to one decimal place. Minor rounding adjustments were made to ensure that all variables total 100%.

## Data Availability

The data presented in this study are available from the corresponding author upon reasonable request due to ethical considerations. The datasets generated and/or analysed during the current study are not publicly available due to ethical constraints on publicising unapproved data. Still, they are available from the corresponding author upon reasonable request.

## Abbreviations

The following abbreviations are used in this manuscript:

COREQ	Consolidated Criteria for Reporting Qualitative Research
ICMR	Indian Council of Medical Research
MHIS	Meghalaya Health Insurance Scheme
NCD	Non-communicable disease
NER	Northeastern Region
OOPE	Out-of-pocket expenditure
OPD	Outpatient department
